# Intelligence Quotient Variability in Klinefelter Syndrome Is Associated With GTPBP6 Expression Under Regulation of X-Chromosome Inactivation Pattern

**DOI:** 10.3389/fgene.2021.724625

**Published:** 2021-09-20

**Authors:** Luciane Simonetti, Lucas G. A. Ferreira, Angela Cristina Vidi, Janaina Sena de Souza, Ilda S. Kunii, Maria Isabel Melaragno, Claudia Berlim de Mello, Gianna Carvalheira, Magnus R. Dias da Silva

**Affiliations:** ^1^Department of Medicine, Escola Paulista de Medicina, Universidade Federal de São Paulo, São Paulo, Brazil; ^2^Department of Biochemistry, Escola Paulista de Medicina, Universidade Federal de São Paulo, São Paulo, Brazil; ^3^Department of Morphology and Genetics, Escola Paulista de Medicina, Universidade Federal de São Paulo, São Paulo, Brazil; ^4^Department of Psychobiology, Escola Paulista de Medicina, Universidade Federal de São Paulo, São Paulo, Brazil

**Keywords:** X-chromosome inactivation, Klinefelter syndrome, X-linked genes, intelligence quotient, GTPBP6, gene expression

## Abstract

Klinefelter syndrome (KS) displays a broad dysmorphological, endocrinological, and neuropsychological clinical spectrum. We hypothesized that the neurocognitive dysfunction present in KS relies on an imbalance in X-chromosome gene expression. Thus, the X-chromosome inactivation (XCI) pattern and neurocognitive X-linked gene expression were tested and correlated with intelligence quotient (IQ) scores. We evaluated 11 KS patients by (a) IQ assessment, (b) analyzing the XCI patterns using both HUMARA and *ZDHHC15* gene assays, and (c) blood RT-qPCR to investigate seven X-linked genes related to neurocognitive development (*GTPBP6*, *EIF2S3*, *ITM2A*, *HUWE1*, *KDM5C*, *GDI1*, and *VAMP7*) and *XIST* in comparison with 14 (male and female) controls. Considering IQ 80 as the standard minimum reference, we verified that the variability in IQ scores in KS patients seemed to be associated with the XCI pattern. Seven individuals in the KS group presented a random X-inactivation (RXI) and lower average IQ than the four individuals who presented a skewed X-inactivation (SXI) pattern. The evaluation of gene expression showed higher *GTPBP6* expression in KS patients with RXI than in controls (*p* = 0.0059). Interestingly, the expression of *GTPBP6* in KS patients with SXI did not differ from that observed in controls. Therefore, our data suggest for the first time that *GTPBP6* expression is negatively associated with full-scale IQ under the regulation of the type of XCI pattern. The SXI pattern may regulate *GTPBP6* expression, thereby dampening the impairment in cognitive performance and playing a role in intelligence variability in individuals with KS, which warrants further mechanistic investigations.

## Introduction

Klinefelter Syndrome (KS) is the most frequent chromosomal disorder in males, with a prevalence of one in 650 newborn males regarding the most common KS karyotype, 47,XXY (Samango-Sprouse et al., 2015, 2019). In addition, this syndrome is associated with endocrinological dysfunctions, in which the individuals present hypogonadism, small and firm testes, gynecomastia, infertility, and some with cognitive impairment (Haltrich, 2019). Although a broad spectrum of phenotypes has been described, KS diagnosis continues to be a challenge, with many patients being misdiagnosed or remain undiagnosed. Currently, karyotype analysis is the most common genetic test for KS diagnosis. Delays in KS diagnosis impair the treatment of patients and the associated comorbidities.

The phenotypic characteristics of patients with KS vary according to the age at diagnosis and different chromosome constitutions, mainly due to mosaicisms. Before puberty, only minor changes are noted, such as slightly reduced testicular volume or elongated lower limbs (Lanfranco et al., 2004; Winge et al., 2020). Many KS individuals present signs of developmental disorders such as dyslexia (Samango-Sprouse, 2001) and attention deficit hyperactivity disorder (Lee et al., 2011). There are also neuropsychiatric conditions, including depression, bipolar disorder, and schizophrenia (Vawter et al., 2007; Giagulli et al., 2019). Behavioral alterations or cognitive dysfunctions are usually detected only at school-age, contributing to the delayed diagnosis of KS.

The Wechsler adult intelligence scale (WAIS-III), used to evaluate overall intelligence, provides a detailed neurocognitive evaluation also for KS (Kaufman et al., 2015). This instrument evaluates the full-intelligence quotient (IQ), as well as verbal (verbal IQ—VIQ), and non-verbal (performance IQ—PIQ) quotients. Individuals with the 47,XXY karyotype usually show average or below-average intelligence performance, with IQs ranging from 80 to 109 (Rovet et al., 1995; Itti et al., 2006; Skakkebaek et al., 2014). The discrepancy between VIQ and PIQ skills, which are considered psychometrically significant variations between standardized scores according to an age range, has not been reported in the literature.

Due to the KS cytogenetic constitution, bearing two X chromosomes, KS patients undergo the X-chromosome inactivation (XCI) process (Raznahan et al., 2018). X-inactivation appears to occur as a mechanism of dosage compensation of the expression of X-linked genes when the cell has more than one X chromosome (Lyon, 1960). This molecular mechanism might play a role in KS phenotype variability (Belling et al., 2017; Raznahan et al., 2018). Moreover, most of the genes on the Y chromosome are homologous to genes on the X chromosome (Mangs and Morris, 2007). These genes are part of the pseudoautosomal regions (PAR1- short arm and PAR2—long arm) and are not subject to X inactivation. However, as widely known, aneuploid cells present unusual gene expression due to the presence of the extra chromosomes, affecting the nuclear architecture (Raznahan et al., 2018; Braun et al., 2019). In the same way, sex chromosome aneuploidies also affect the expression of X-linked genes that escape from XCI (Zhang et al., 2020). For these reasons, the expression pattern of seven genes associated with cognition (*GTPBP6*, *EIF2S3*, *ITM2A*, *HUWE1*, *KDM5C*, *GDI1*, and *VAMP7*)(Vawter et al., 2007; Gecz et al., 2009; Tuttelmann and Gromoll, 2010; Lagha et al., 2013; Piton et al., 2013; Belling et al., 2017; Gravholt et al., 2018) and *XIST* was chosen as a strategic analysis to understand, at least in part, the phenotypic variability in KS (Skakkebaek et al., 2018).

We aimed to assess whether the intelligence performance variability of KS patients is associated with the X inactivation pattern and expression profile of X-linked genes. Our study regarding XCI and IQ analyzed individuals presenting karyotype 47,XXY and considered them in statistical comparisons with male 46,XY and female 46,XX controls. We also describe the findings observed in individuals presenting the 49,XXXXY, and 48,XXYY karyotypes.

## Subjects and Methods

### Cytogenetic Analysis

We enrolled 27 subjects, 11 KS patients (mean age 31.7 years; SEM 4.3) and two polysomic individuals (49,XXXXY, P12; 48,XXXY, P13), from the endocrinology outpatient clinic of University’s Hospital, as indicated in Table 1, as well as seven female (mean age 37.3 years; SEM 6.0) and seven male (mean age 35.3 years; SEM 4.3) healthy controls. The 550-resolution G-banding chromosomal analysis at a minimum of 20 lymphocyte metaphases confirmed karyotypes. In addition, we performed assays to study the XCI pattern in the patients and the mothers from three patients (P3, P5, and P12) who agreed to participate in the study. All patients signed informed consents after a fully explanating of the purpose and nature of all procedures used, which the University Ethics Committee had approved (CEP/Unifesp: 1.774.406).

**TABLE 1:** Sociodemographic, clinical, and neuropsychological characterization and X-chromosome inactivation features observed in Klinefelter syndrome.

**Feature/patient (P)**	**Klinefelter syndrome (47,XXY)**											**Polysomic (49,XXXXY and 48,XXYY)**		**Mean (SD)**
	**P1**	**P2**	**P3**	**P4**	**P5**	**P6**	**P7**	**P8**	**P9**	**P10**	**P11**	**P12**	**P13**	
**WAIS-III general**														
Age at diagnosis	8	18	19	15	18	13	24	53	47	17	38	5	16	22.38 (14.6)
Age at psychological and XCI pattern analysis	24	32	22	26	17	15	35	62	49	26	41	24	22	30.4(13.4)
Schooling (y)	0	2	0	0	8	8	11	4	4	12	11	0	11	5.5 (4.5)
Comorbidity	Epi	Epi	No	Epi^§^	No	No	No	No	Ob	No	No	DM	SCZ	–
Employment (Y/N)	N	N	N	Y	N	N	Y	N	Y	Y	Y	N	N	–
Full-scale IQ	66	70	71	74	77	81	99	80	87	99	109	<55^*^	80	80.6 (13.3)
VIQ	66	70	68	71	74	80	l100	77	86	89	101	NA	80	80.3 (11.7)
PIQ	68	72	77^&^	81^&^	81	84	99	87^&^	90	111^&^	118^&^	NA	83	87.6 (15.0)
FSIQ classification	EL	B	B	B	B	LA	A	LA	LA	A	A	EL	LA	87.6 (15.0)

**WAIS subtest analysis**														
**verbal**														
Vocabulary	1	3	4	3	3	5	7	3	6	8	10	Δ	4	4.7 (2.6)
Similarities	6	5	5	6	2	5	10	9	8	10	11	Δ	8	7.3 (2.3)
Arithmetic	4	6	5	5	8	11	10	4	8	8	10	Δ	6	7.1 (2.4)
Digit span	5	6	4	4	7	5	12	8	8	7	11	Δ	5	6.8 (2.6)
Information	5	6	6	7	4	5	11	8	8	8	10	Δ	11	7.7 (2.1)
Comprehension	4	3	3	5	5	6	10	4	7	8	9	Δ	5	5.8 (2.3)
**performance**														
Figure completion	6	4	6	8	5	10	9	9	10	13	14	Δ	4	8.2 (3.3)
Coding	2	5	6	10	10	7	10	9	8	10	10	Δ	8	7.9 (2.5)
Block design	5	6	6	6	6	7	11	7	9	11	11	Δ	5	7.6 (2.4)
Matrix reasoning	5	7	6	5	5	8	10	7	8	13	16	Δ	7	8.3 (3.3)
Picture arrangement	6	5	7	5	6	5	9	7	7	12	14	Δ	12	7.9 (3.1)

**Molecular assay**														
**Ratio**														
HUMARA	HMZ	HMZ	37/63	42/58	HMZ	68/32	HMZ	26/74	HMZ	30/70	75/25	43/57	75/25	–
*ZDHHC15*	54/46	54/46	–	–	47/53	51/49	52/48	–	16/84	–	–	–	–	–
**XCI**														
Pattern	RXI	RXI	RXI	RXI	RXI	RXI	RXI	SXI	SXI	SXI	SXI	RXI	SXI	–

Wechsler’s Adult Intelligence Scale (WAIS-III) assesses the global Intelligence Quotient (Full-scale IQ), as well as intelligence score resulting from verbal (Verbal IQ) and non-verbal (Performance IQ) domains. WAIS-III is further composed of 13 subtests that measure specific cognitive abilities. We used 11 of these subtests to analyze only Verbal IQ (vocabulary, similarities, arithmetic, digit span, information, and comprehension) and Performance IQ (picture completion, digit symbol, block design, matrix reasoning, and picture arrangement). Schooling is related to the total time in the school, including failure results. IQ, Intelligence Quotient. VIQ, verbal intelligence quotient. PIQ, performance intelligence quotient. NA, not applicable. Y, yes; N, no. (*) converted from percentile 1. (&), significant difference between VIQ and PIQ scores. (Δ), who did not understand WAIS-III instructions. §, history of seizures in childhood. Epi, Epilepsy. Ob, Obesity. DM, Diabetes mellitus. SCZ, Schizophrenia. No, nothing reported. HMZ, homozygous. Ratio, minor/major allele proportion using either, androgen receptor (AR) or zinc finger DHHC-type containing 15 (ZDHHC15) polymorphisms. RXI, random X-inactivation. SXI, skewed X-inactivation.

### Intelligence Quotient Assessment

The intelligence quotient (IQ) of the patients was assessed using the Brazilian version of the Wechsler Adult Intelligence Scale—WAIS-III (Bowden et al., 2006). A trained neuropsychologist undertook the process. WAIS-III assesses the overall intelligence quotient (full-scale IQ), as well as intelligence associated with verbal (verbal IQ) and non-verbal (performance IQ) domains. Verbal IQ (VIQ) is a measure of verbal reasoning and acquired knowledge, including social rules. Performance IQ (PIQ), on the other hand, is established by tasks that measure non-verbal reasoning, spatial processing, and visual-motor integration (Lange, 2011). WAIS-III is composed of 13 subtests that measure specific cognitive abilities linked to four factor-based indices: verbal comprehension (vocabulary, similarities, and information), perceptual organization (picture completion, block design, and matrix reasoning), working memory (arithmetic, digit span, and letter-number sequencing), and processing speed (digit symbol, and symbol search). The main IQ and Wechsler scale indexes are expressed as standard scores (100 ± 15) and the subtests as scaled scores (10 ± 3). The IQ ranges and classifications are: ≥ 130: very superior; 120–129: superior; 110–119: high average; 90–109: average; 80–89: low average; 70–79: borderline; ≤ 69: extremely low.

### RNA Isolation and Quantitative RT-PCR

The expression level of seven X-chromosome genes *EIF2S3*, *GDI1*, *ITM2A*, *GTPBP6*, *HUWE1*, *KDM5C*, *VAMP7* (Supplementary Table 1), and *XIST* was quantified by quantitative RT-PCR in the 11 KS patients, 2 polysomic patients and 14 (male and female) controls. Total RNA was isolated from peripheral blood using Trizol^®^ reagent, according to the manufacturer’s instructions (Invitrogen Corp., Carlsbad, CA). One microgram of total RNA from each sample was reverse transcribed into cDNA using oligo (dT) primers. cDNA was then diluted fivefold, and 1.0 μL aliquot of cDNA was used in a 12 μL PCR reactions containing SYBR Green PCR Master Mix (Applied Biosystems, Foster City, CA) and 10 μmol/L of each primer for target gene or reference gene (*RPS8*). PCR primers are listed in Supplementary Table 2.

RT-qPCR reactions were performed in triplicate, and the threshold cycle (Ct) was averaged (SD ≤ 1). The fold changes were calculated according to the comparative ΔΔCt method using *RPS8* as reference gene, as described by Carvalheira et al. (2015). The male individuals were used as a reference group. Alternatively, for the correlation test, we represented gene expression as 1/ΔCt with no normalization to a control group. Higher 1/ΔCq values indicate higher gene expression.

### Human Androgen Receptor Assay (HUMARA)

XCI assays were performed in 16 individuals, comprising 11 karyotyped 47,XXY KS patients (P1-11), two patients with karyotypes 49,XXXXY (P12) and 48,XXYY (P13), and three mothers of from the patients P3, P5 and P12. The HUMARA assay was performed according to the manufacturer’s protocol as previously described (Sisdelli et al., 2016). Two primer pairs were designed to the first exon of the androgen receptor (*AR*) gene, located at Xq11.2. However, when the results were non-informative for the polymorphic region of the AR gene, another primer pair was designed to amplify the polymorphic region of the zinc finger DHHC-type containing 15 (*ZDHHC15*) gene, located at Xq13.3, as previously described (Bertelsen et al., 2011). Primers are listed in Supplementary Table 2. All reactions were performed in triplicate, except for patient 12, which were carried out in quintuplicate.

The pattern of XCI was determined by the equation proposed by Bolduc et al. (2008), which is used to obtain the percentage of a digested allele with the greatest number of CAG_n_ repeats. The undigested samples were used as both the control of the digested reactions and to calculate the number of CAG_n_ repeats. The XCI was considered random when the ratio presented values between 50 and 65%, indeterminate between 66 and 73%, skewed when between 74 and 90%, and extremely skewed between 90 and 100% (Bolduc et al., 2008).

### Statistical Analysis

Intellectual performance measures were analyzed descriptively. Intelligence quotient scores and relative gene expression levels meeting parametric criteria were expressed as means, and the two-tailed Student’s *t*-test was applied for two group comparisons, while one-way ANOVA followed by Bonferroni test was performed for multiple group comparisons. Non-parametric data were expressed as median, and Kruskal-Wallis followed by Dunn test was used for multiple group comparisons. Pearson’s correlation coefficient (r) was calculated to evaluate the correlation between two numeric variables. GraphPad© PRISM (Version 8.0; San Diego, CA) was used for all statistical analyses. A *P*-value of < 0.05 was considered statistically significant. Polysomic patients were not considered in the statistical comparison of gene expression between KS (47,XXY) patients and controls due to their phenotypic and molecular variability (Braun et al., 2019).

## Results

### Klinefelter Patients Present a Broad Intelligence Quotient Variability

Applying the Wechsler Adult Intelligence Scale with age-related assessment criteria, we verified that 11 KS patients with karyotype 47,XXY displayed total IQ values varying from 66 to 109, which correspond to extremely low to average levels a broad range of variation of the intelligence performance in our cohort. Sociodemographic, clinical and neuropsychological characterization, including the schooling, clinical comorbidity, VIQ (Vocabulary, Similarities, Arithmetic, Digit Span, Information and Comprehension), and PIQ (Figure Completion, Coding, Block Design, Matrix Reasoning, and Picture Arrangement), are described in Table 1. There was a difference of at least nine points between the PIQ and VIQ scores in five (P3, P4, P8, P10, and P11) individuals, with a better performance in the former. Regarding polysomic participants, the patient with the variant karyotype 49,XXXXY (P12) presented a severe intellectual disability, score < 1st percentile in the Raven’s Progressive Matrices Scale, corresponding to an IQ ≤ 55, while the patient with karyotype 48,XXYY (P13) showed a low average level IQ.

### The Skewed X Inactivation Pattern in the Blood Is Associated With a Better Intelligence Quotient Score

To assess whether the XCI pattern is associated with the intelligence phenotype of KS patients, we used HUMARA and *ZDHHC15* assays. Among the KS patients, seven (64%) demonstrated random X inactivation (RXI), while four (36%) presented skewed X inactivation (SXI) (Table 1). The mean ages at KS diagnosis and XCI pattern analysis were, respectively, 16.4 years (SEM 1.9) and 24.4 years (SEM 2.8) for patients with RXI and 38.8 years (SEM 7.9) and 44.5 (SEM 7.5) for patients with SXI pattern. In addition, the two polysomic patients with 49,XXXXY and 48,XXYY karyotypes showed RXI (P12) and SXI (P13), respectively (Table 1).

**FIGURE 1:**
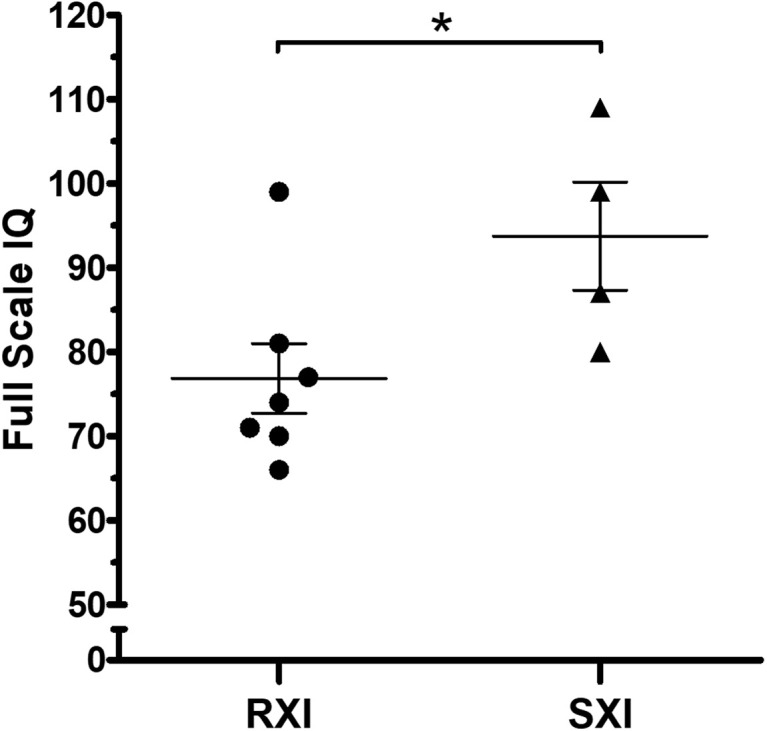
Intelligence quotient scores in Klinefelter syndrome according to the X-chromosome inactivation pattern. RXI patients (*n* = 7) show a lower IQ mean than SXI patients (*n* = 4). The mean IQ from patients with RXI was at borderline levels (Mean = 76.86), while in the SXI patients, it was average (Mean = 93.75). Data are presented as mean ± SEM. Statistical analysis used the *t*-test (*p = 0.0451). IQ, intelligence quotient. RXI, random X-chromosome inactivation; SXI, skewed X-chromosome inactivation.

The mean IQ of the seven patients with RXI was at borderline levels (Mean = 76.86), while for the four patients with SXI, it was average (Mean = 93.75), demonstrating that in this cohort, SXI is present in KS patients with higher IQ values (Figure 1, *p* = 0.0451). Four patients with borderline IQ levels (P2, P3, P4, and P5), ranging from 70 and 77, presented RXI (Table 1). One patient (P1) with intellectual deficiency (IQ 66) presented RXI. On the other hand, four patients with low to average IQ levels (P8, P9, P10, and P11), ranging from 80 to 109, presented an SXI pattern. The polysomic patients also followed the association between XCI and IQ. P12 (49,XXXXY) presented an IQ ≤ 55 and RXI, while P13 (48,XXYY) presented an IQ of 80 and SXI (Table 1).

Based on this broad IQ variability, we further tested whether seven X-chromosome genes, previously associated with neurocognitive function, would be altered in expression in KS patients and whether the expression of these genes was correlated with XCI patterns.

### The X-Inactivation Pattern Interferes in GTPBP6 Expression, a Gene Related to Intellectual Performance

**FIGURE 2:**
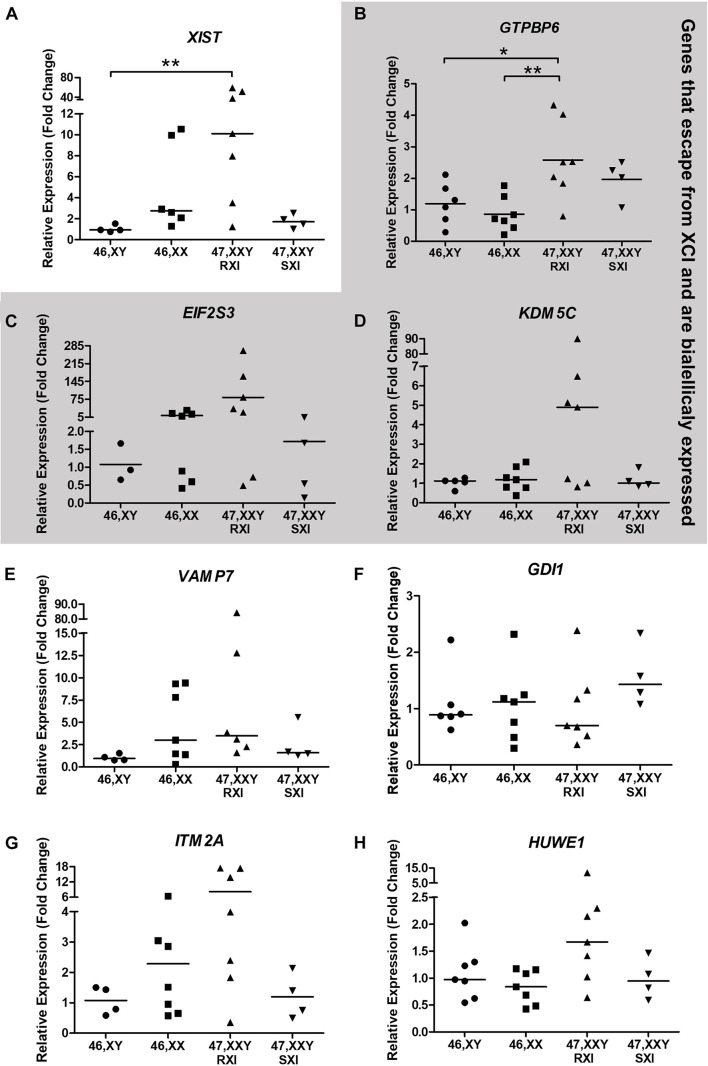
Neurocognitive-related X-chromosome linked gene expression analysis tested in Klinefelter Syndrome patients in comparison to healthy controls. Eight X-linked genes, **(A)** *XIST*, **(B)** *GTPBP6*, **(C)** *EIF2S3*, **(D)** *KDM5C*, **(E)** *VAMP7*, **(F)** *GDI1*, **(G)** *ITM2A*, and **(H)** *HUWE1*, were evaluated. Genes that escape from X-chromosome inactivation and are biallelically expressed are indicated in the gray area. Relative expression (fold change) is represented in relation to males (XY). Note that in **(A)**, the *XIST* expression was higher in KS patients with RXI in comparison to male controls (*p* = 0.0090), while in **(B)**, the *GTPBP6* expression was higher in KS patients with RXI than in both female and male controls (*p* = 0.0059). In **(A,C,D,E,F),** data are presented as the median and the statistical test used was Kruskal-Wallis followed by the Dunn test. In **(B,G,H)**, data are presented as the mean, and statistical analysis used was one-way ANOVA followed by the Bonferroni test. *p < 0.05; **p < 0.01. The number of individuals considered per group is indicated in Supplementary Table 3.

In respect of the general expression analysis of the eight selected X-linked genes, *GTPBP6*, *EIF2S3*, *ITM2A*, *HUWE1*, *KDM5C*, *XIST*, *GDI1*, and *VAMP7*, only *XIST* (Xq13.2) as expected, and the pseudoautosomal gene *GTPBP6* (Xp22.33/Yp11.32) showed differential expression (Figure 2). *XIST* expression was higher in KS patients with RXI than in male controls (*p* = 0.0090, Figure 2A). KS patients with the RXI pattern presented higher *GTPBP6* expression when compared with both male and female controls (*p* = 0.0059). Interestingly, the expression of *GTPBP6* in KS patients with SXI did not differ from that observed for male and female controls (Figure 2B). Also, the four KS patients with the SXI pattern presented general expression level profiles similar to the male controls (Figure 2). The gene expression levels for the polysomic patients are presented in Supplementary Table 3 for comparison.

**FIGURE 3:**
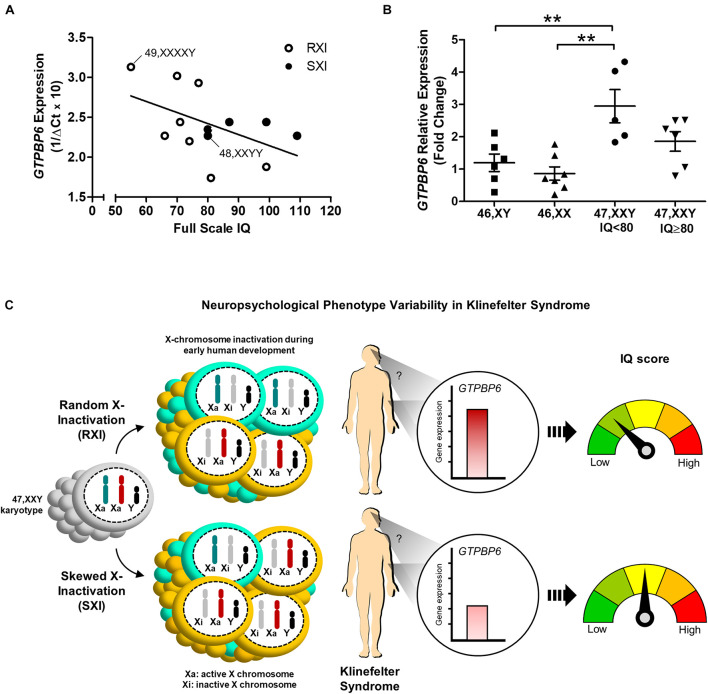
Intelligence quotient variability in association with the X-chromosome inactivation pattern and *GTPBP6* gene expression evidenced in Klinefelter syndrome patients. **(A)** Pearson’s correlation between *GTPBP6* expression and full-scale intelligence quotient in Klinefelter patients. *GTPBP6* expression was calculated as 1/ΔCq using *RPS8* as the reference gene. Higher 1/ΔCq values indicate greater *GTPBP6* expression. Linear regression was calculated (slope = –0.01385). Klinefelter and polysomic patients with RXI and SXI are represented with white and black circles, respectively. Correlation *r* = –0.5045, *p* = 0.0787. Polysomic subjects are indicated. **(B)** Peripheral blood *GTPBP6* gene expression analysis in KS patients with IQ below the standard average of 80 (*n* = 5) in comparison to both male (XY) and female (XX) controls. All KS patients with IQ < 80 presented a random X-inactivation pattern (RXI). Relative expression (fold change) is represented in relation to males. Note that *GTPBP6* expression was higher in KS patients with IQ < 80 in comparison to both male and female controls (*p* = 0.0012). Data are presented as mean ± SEM and statistical analysis used One-way ANOVA followed by Bonferroni test (**p < 0.01). **(C)** Hypothetical model for understanding of the correlation between intelligence quotient, X-inactivation pattern and altered *GTPBP6* expression in Klinefelter patients. This model suggests that the intelligence quotient in KS individuals may reflect the X-inactivation pattern. Also, when presenting RXI, the *GTPBP6* expression may be elevated and the intelligence quotient is low. On the other hand, when presenting the SXI pattern, the *GTPBP6* expression is low and the intelligence quotient is average. Our data indicate that the SXI pattern may downregulate *GTPBP6* expression, leading to a better cognitive performance in KS patients. Color schemes for the XCI patterns show 50% of the cells with the same Xi representing RXI, and 75% of the cells with the same Xi representing SXI, following the classification in Bolduc et al. (2008). IQ, intelligence quotient; RXI, random X-inactivation; SXI, skewed X-inactivation.

We further investigated whether *GTPBP6* expression would correlate to intelligence assessment. Although Pearson’s correlation test suggested a negative correlation trend between *GTPBP6* expression and full-scale IQ (*r* = –0.5045), it was not statistically significant (*p* = 0.0787) among all KS and polysomic patients (Figure 3A). However, when considering IQ 80 as the standard minimum reference, five patients (45%) with IQ < 80 presented the RXI pattern and showed higher *GTPBP6* expression (Mean = 2.946) when compared with both female and male controls (*p* = 0.0012) (Figure 3B). On the other hand, all the SXI patients presented IQ ≥ 80, with four classified as SXI, and showed *GTPBP6* expression levels that did not differ from controls (Figure 3B).

## Discussion

Although there has been significant progress in understanding genetics concerning neurodevelopmental disorders over the last two decades, little is known about the etiopathogenesis of cognitive impairment in 47,XXY patients. Typically, each second X-chromosome is silenced in half of the cells randomly, but this pattern may be skewed when preferential inactivation of an X-chromosome occurs. Therefore, we hypothesized that the X-inactivation pattern might interfere with cognitive phenotype development in KS. To address this topic, we assessed the XCI patterns as well as the expression of seven X-linked genes associated with neurocognitive function and their correlation with intelligence performance in KS patients.

Our data endorsed the variability in intelligence performance in the typical 47,XXY KS cohort, as reported previously (Samango-Sprouse, 2001; Tartaglia et al., 2010; Brandenburg-Goddard et al., 2014; Skakkebaek et al., 2014; Samango-Sprouse et al., 2018). We also verified that VIQ was significantly lower than PIQ in KS patients with average IQ, which has been known that lower VIQ is associated with language impairments, commonly observed in KS (Gravholt et al., 2018). Higher PIQ scores indicate that non-verbal skills (e.g., analyzing and synthesizing visual stimuli) are well developed. In other words, it seems that verbal tasks (word definition, verbal comprehension, concept formation, and the ability to define concepts orally) may be incredibly challenging for some KS patients. It is important to note that intellectual disability (IQ < 70) is rare in KS patients, and that verbal intellectual skills (VIQ) are usually more affected than non-verbal (PIQ) skills (Marco and Skuse, 2006; Skakkebaek et al., 2014; Melogno et al., 2018). A similar intellectual profile (VIQ < PIQ) is also observed in autism spectrum disorder (Ankenman et al., 2014).

Intellectual disability was detected in one and a borderline level IQ in four cases. Although intellectual variability in KS has already been described (Samango-Sprouse, 2001; Tartaglia et al., 2010; Skakkebaek et al., 2014; Brandenburg-Goddard et al., 2014; Samango-Sprouse et al., 2018), reports of disability are not usual. A high neurocognitive phenotypic variability implies that KS condition is frequently underdiagnosed. Consequently, hypogonadic individuals might not receive early hormonal replacement treatment, which may affect cognitive performance (Samango-Sprouse et al., 2013, 2015). It is also possible that the wide variation of IQ identified in our sample may be because the patients referred to our service, which is a tertiary reference center, usually present more complex clinical manifestations.

In KS, comorbidities such as an increased risk for obesity, diabetes, and seizures have been reported (Belling et al., 2017). A predisposition to metabolic diseases has been associated with a possible biological function of the methylation changes found in KS, as demonstrated by gene expression enrichment analysis (Skakkebaek et al., 2018). Diabetes, epilepsy, schizophrenia, or obesity was present in almost half (46%) of our patients. Some of these clinical conditions can negatively impact cognitive development and may have contributed to the intellectual variability (Al-Shazely and Al-Khaligy, 2014). A high rate of epilepsy has been reported in patients with other sex chromosome aneuploidies (Tatum et al., 1998; Verri et al., 2017). Noteworthy, P1 (47,XXY) and P12 (49,XXXXY) presented complex phenotypic variation besides epilepsy, including autoimmune disease (Churg-Strauss syndrome) and refractory controlling seizures (P1).

Many studies have attempted to establish criteria for early diagnosis of KS (Belling et al., 2017), with chromosome karyotype analysis being the best method of diagnostic confirmation so far. The phenotypic variability seems to result from both genetic and environmental factors, although the relative contribution of these events remains unclear. Regarding genetic influences, variability appears to be associated with a gene dosage effect due to the additional X chromosome. It can be the result of X-inactivation (Iitsuka et al., 2001), uniparental disomy of the sex chromosomes (Bruining et al., 2010), and a positive family history of learning disabilities (Samango-Sprouse et al., 2013, 2014).

As is widely known, XCI occurs in cells that contain more than one X chromosome, which increases the chance of survival of male aneuploid KS individuals compared to those with other forms of aneuploidy. XCI occurs due to the high expression of the non-coding RNA *XIST*, upregulated in 46,XX subjects (Vawter et al., 2007; Minks et al., 2008; Belling et al., 2017). This genetic phenomenon interferes with the X-linked genes’ expression and results in symptoms that may vary in individuals according to the XCI pattern. It has been suggested that SXI in KS patients occurs as a common genetic event (Iitsuka et al., 2001). However, it has also been shown that 10–50% of KS patients present SXI (Amos-Landgraf et al., 2006; Bojesen et al., 2011; Mehta et al., 2012; Kinjo et al., 2020). Our data show similar results to these studies. Seven patients (64%) presented the RXI pattern, and four (36%) the SXI pattern. It is known that 5–20% of seemingly healthy 46,XX normal subjects also display SXI (Belmont, 1996). It has been reported that females who carried out X-linked recessive lethal allele, or an allele that result in a proliferative advantage for cells, show skewing of X-inactivation (Naumova et al., 1996). These observations suggest that the XCI pattern in somatic cells may be influenced by the X-chromosome genetic background, promoting a cellular selection favoring the best genetic balance (Gartler and Sparkes, 1963). Another molecular mechanism involved in the XCI pattern is aging (Sharp et al., 2000; Amos-Landgraf et al., 2006). The prevalence of SXI is higher in older than in younger women (Sandovici et al., 2004). Patients with RXI pattern had a mean age at KS diagnosis and XCI pattern analysis lower than with SXI. These data may reflect that the RXI pattern could enhance the severity of the symptoms, while in KS patients with SXI, the clinical features could be mild, including those related to neurocognitive performance.

Another genetic feature related to the extra sex chromosome observed in the KS is the trisomy of pseudoautosomal genes (Zhang et al., 2013; Carrel and Brown, 2017; Balaton et al., 2018). These events would explain clinical symptoms and the susceptibility to comorbidities. The excess of gene expression from one or more X-linked genes that escape inactivation could be responsible for the cognitive phenotype in psychotic disorders diagnosed in KS (DeLisi et al., 2005; Wallentin et al., 2016), the reason by which we analyzed the expression of seven X-linked genes involved in neurodevelopment collected from peripheral blood cells. Indeed, a robust RNA-seq-based study has demonstrated that an XCI pattern from the blood is predictive of X-inactivation status in other tissues, such as the brain (Tukiainen et al., 2017), although these tissues have a different embryonic origin.

We demonstrated that GTP Binding Protein 6 (*GTPBP6*) gene presented differential expression among the other neurocognitive X-linked genes in KS studied group. *GTPBP6* is expressed in the fetal and adult brain and has been negatively correlated with language functioning (Gianfrancesco et al., 1998; Vawter et al., 2007). Noteworthy, large-scale human exome sequencing data have found mutations in other GTPases (Rho family), causing X-linked intellectual disability (Piton et al., 2013). The overexpression of this gene was reported in lymphoblastoid cell lines derived from 11 males with KS, among 14 differentially expressed X-chromosome genes in a KS group (Vawter et al., 2007). Our results complement these data, demonstrating that higher *GTPBP6* expression is observed in KS patients with RXI but not with SXI. This observation suggests that epigenetic events under the X-chromosome inactivation process may somehow interfere with the regulation of the genes involved in cognition in KS.

Moreover, our data suggested that *GTPBP6* expression was inversely correlated with IQ scores in KS patients. On the other hand, KS patients with *GTPBP6* expression similar to controls presented SXI patterns and higher IQ scores than those with RXI. This finding indicates that the SXI pattern somehow stabilizes *GTPBP6* expression at levels similar to controls, which may function as a protective factor for cognitive development. Furthermore, it is known that in individuals with sex-chromosome aneuploidies even genes that escape X-inactivation are under the influence of the epigenetic events participating in this molecular mechanism, such as neighboring DNA methylation and changes in topologically associating domain (TAD) boundaries (Raznahan et al., 2018; Braun et al., 2019). Thus, these data corroborate the idea of *GTPBP6* as a non-inactivated gene reflecting the XCI pattern. *GTPBP6* is among the most sensitive genes to sex chromosome dosage (Raznahan et al., 2018), which may be reflected in the range of cellular and molecular strategies to regulate its expression in KS. These underlying molecular mechanisms in humans are still poorly understood and may involve complex signaling pathways that are yet to be investigated.

It is important to note that SXI may reflect selection against uniparental disomy and gene dosage, favoring cell survival and viability. Studies using murine models demonstrated a global increase in DNA methylation on the X chromosome from the paternal origin compared to the maternal one, implicating higher expression of X genes in XY than XX cells (Golden et al., 2019). In KS patients, neurocognitive functions were reported to be influenced by the parental origin of the extra X chromosome (Navarro-Cobos et al., 2020). Considering the data presented here, it is likely that the SXI pattern may be a molecular mechanism that guarantees cellular homeostasis. Thus, the effects of sex chromosome trisomy can be dampened, and, consequently, the genes present in the pseudoautosomal region, such as *GTPBP6*, would have their expression regulated by preferential inactivation of the X-chromosome.

Therefore, dysregulation of X-linked genes seems to cause an epigenetic imbalance that explains most of the changes observed in sex chromosome aneuploidies, particularly in KS. Our findings are summarized in Figure 3C, in which we propose a hypothetical model for understanding the correlation between X-inactivation pattern and altered *GTPBP6* expression, being inversely related to intelligence performance. This model implicates the SXI pattern as a protective molecular strategy mitigating the deleterious effects of unbalanced gene expression related to the extra X chromosome. These data shed light on the contribution of the XCI pattern and *GTPBP6* expression to the neurocognitive phenotype observed in KS patients and may help to elucidate the molecular mechanisms associated with neurodevelopment dysfunction in X-linked aneuploid disorders.

## Data Availability Statement

The datasets analyzed for this study will be found in the Unifesp Repository (http://repositorio.unifesp.br/).

## Ethics Statement

The studies involving human participants were reviewed and approved by the Universidade Federal de São Paulo (Unifesp) Ethics Committee N° 1.774.406. Written informed consent to participate in this study was provided by the participants’ legal guardian/next of kin.

## Author Contributions

LS assisted patients and performed all neuropsychological tests. LF, AV, JS, and IK performed molecular and bioinformatics analysis. MM and GC reviewed cytogenetics and gene expression data. LS, LF, CM, GC, and MD designed the study, performed, and reviewed statistics. GC and MD equally conceived and coordinated the study, edited, and reviewed the manuscript. All authors discussed the data and contributed to writing the final manuscript.

## Conflict of Interest

The authors declare that the research was conducted in the absence of any commercial or financial relationships that could be construed as a potential conflict of interest.

## Publisher’s Note

All claims expressed in this article are solely those of the authors and do not necessarily represent those of their affiliated organizations, or those of the publisher, the editors and the reviewers. Any product that may be evaluated in this article, or claim that may be made by its manufacturer, is not guaranteed or endorsed by the publisher.
