# Supplementary material for: Intelligence Quotient Variability in Klinefelter Syndrome Is Associated With GTPBP6 Expression Under Regulation of X-Chromosome Inactivation Pattern
Source: Front Genet. 2021 Sep 20;12:724625. doi: 10.3389/fgene.2021.724625 (PMC8488338; doi:10.3389/fgene.2021.724625)
Supplement: Supplementary file 1 [file Data_Sheet_1.docx]

**Supplementary data**

**Simonetti et al.**

| **Supplementary Table S1.** X-chromosomal genes related to intellectual performance (excepting *XIST*) selected for expression analysis. | | | | |
| --- | --- | --- | --- | --- |
| **Gene symbol** | **OMIM** | **Name** | **Chromosome Region (GRCh37/hg19)** | **Escape from XCI** |
| *GTPBP6* | 300124 | GTP-Binding Protein 6 | Xp22.33/Yp11.32 | Yes |
| *EIF2S3* | 300161 | Eukaryotic Translation Initiation Factor 2, Subunit 3 | Xp22.11 | Yes |
| *HUWE1* | 300697 | HECT, UBA, and WWE domains-containing protein 1 | Xp11.22 | No |
| *ITM2A* | 300222 | Integral Membrane Protein 2A | Xq21.1 | No |
| *KDM5C* | 314690 | Lysine-specific Demethylase 5C | Xp11.22 | Yes |
| *XIST* | 314670 | X Inactivation-Specific Transcript | Xq13.2 | Yes |
| *GDI1* | 300104 | GDP Dissociation Inhibitor 1 | Xq28 | No |
| *VAMP7* | 300053 | Vesicle-Associated Membrane Protein 7 | Xq28/Yq12 | No |
| X-linked genes were selected based on their previously reported involvement with intellectual performance, except for *XIST* (Vawter et al., 2007; Gécz et al., 2009; Tüttelmann and Gromoll, 2010; Lagha et al., 2013; Piton et al., 2013; Belling et al., 2017 and Gravholt et al., 2018) . XCI, X-chromosome inactivation. | | | | |
|  |  |  |  |  |

|  | | | | |
| --- | --- | --- | --- | --- |
| **Supplementary Table S2.** Oligonucleotide sequence list used in the study for X-chromosome inactivation and gene expression | | | | |
| **Assay** | **Primer** | **5'- 3' Sequence** | **Product size (bp)** | **RefSeq**  **(NCBI)** |
| **X-chromosome Inactivation** |  |  |  |  |
| AR_First PCR | *Forward 1* | GGGTAAGGGAAGTAGGTGGAAG | 996 | NG_009014.2 |
|  | *Reverse 1* | CCTTTGGTGTAACCTCCCTTGA |  |  |
| AR_Nested PCR | *Forward 2* | FAM-GTGCGCGAAGTGATCCAGAA | 244 |  |
|  | *Reverse 2* | CACAGGCTACCTGGTCCTGG |  |  |
| *ZDHHC15* | *Forward* | FAM-TCTTTGGCTCGAAGATCGAC | 539 | NG_012974.1 |
|  | *Reverse* | TATGGCTCGCATCTTTCACA |  |  |
| **Gene expression** |  |  |  |  |
| *GTPBP6* | *Forward* | GGGGAGACAGATCCTCACTCT | 93 | NM_012227.3 |
|  | *Reverse* | ATCACGTCCACCTCCTGAAC |  |  |
| *EIF2S3* | *Forward* | TTTTGAGACGGAGGCTCACT | 109 | NM_001415.3 |
|  | *Reverse* | GAGGCTGAAGCACGAGAATC |  |  |
| *HUWE1* | *Forward* | TCGAGATGACAGGTCCACAG | 80 | NM_031407.6 |
|  | *Reverse* | AGCTCTCATAGGCAGGCAGA |  |  |
| *ITM2A* | *Forward* | CAGAAAGTCCTTCCGCCTTC | 100 | NG_016412.1 |
|  | *Reverse* | AAATTCGTTGGGGAAGTGTC |  |  |
| *KDM5C* | *Forward* | GAGGTGACCCTGGATGAGAA | 90 | NM_001146702.1 |
|  | *Reverse* | CCAGGAGCTGAGGTCTGAAC |  |  |
| *XIST* | *Forward* | TGAGTCTTTGCTGTTTGGAAGA | 109 | NR_001564.2 |
|  | *Reverse* | CCAACTCCCCAGTTTGTTTC |  |  |
| *GDI1* | *Forward* | GCCCATTGACCAGAAGTTTG | 114 | NM_001493.2 |
|  | *Reverse* | AAGTGTGTGGTGGCATCGTA |  |  |
| *VAMP7* | *Forward* | GCTCGAGCCATGTGTATGAA | 114 | NM_001145149.2 |
|  | *Reverse* | GCTTGGCCATGTAAATCCAC |  |  |

| **Supplementary Table S3.** Comparison between gene expression categorized by the X-inactivation pattern and intelligence quotient. | | | | | | | |
| --- | --- | --- | --- | --- | --- | --- | --- |
|  |  | **Male (46,XY)** | **Female (46,XX)** | **KS**  **(47,XXY)** | **KS (47,XXY)** | **P12 (49,XXXXY)** | **P13 (48,XXYY)** |
|  |  |  |  | **RXI** | **SXI** | **RXI** (n=1) | **SXI** (n=1) |
| **IQ value** |  |  |  | 76.86 ± 4.13 | 93.75 ± 6.42 | < 55 | 80 |
|  |  |  |  | (n=7) | (n=4) |  |  |
| **Normalized gene expression levels  in relation to males (46,XY)** | ***XIST*** | 1.04 ± 0.17 | 4.90 ± 1.71 | 24.56 ± 9.26 | 1.74 ± 0.32 | 5.82 | 3.89 |
|  |  | (n=4) | (n=6) | (n=7) | (n=4) |  |  |
|  | ***ITM2A*** | 1.08 ± 0.23 | 2.29 ± 0.79 | 8.16 ± 2.90 | 1.20 ± 0.37 | 2.71 | 2.07 |
|  |  | (n=4) | (n=7) | (n=7) | (n=4) |  |  |
|  | ***HUWE1*** | 1.09 ± 0.19 | 0.83 ± 0.12 | 3.01 ± 1.50 | 0.99 ± 0.19 | 0.61 | 1.58 |
|  |  | (n=7) | (n=7) | (n=7) | (n=4) |  |  |
|  | ***KDM5C*** | 1.03 ± 0.12 | 1.19 ± 0.23 | 15.65 ± 12.42 | 1.17 ± 0.22 | 1.38 | 5.75 |
|  |  | (n=5) | (n=7) | (n=7) | (n=4) |  |  |
|  | ***GDI1*** | 1.09 ± 0.23 | 1.06 ± 0.25 | 1.02 ± 0.26 | 1.57 ± 0.28 | 0.34 | 0.78 |
|  |  | (n=6) | (n=7) | (n=7) | (n=4) |  |  |
|  | ***VAMP7*** | 1.04 ± 0.18 | 4.66 ± 1.52 | 17.98 ± 13.37 | 2.51 ± 1.02 | 0.33 | 1.40 |
|  |  | (n=4) | (n=7) | (n=6) | (n=4) |  |  |
|  | ***EIF2S3*** | 1.08 ± 0.30 | 11.09 ± 4.49 | 82.33 ± 37.62 | 1.72 ± 0.99 | 6.16 | 6.94 |
|  |  | (n=3) | (n=7) | (n=7) | (n=4) |  |  |
|  | ***GTPBP6*** | 1.19 ± 0.27 | 0.86 ± 0.21 | 2.58 ± 0.47 | 1.96 ± 0.31 | 4.72 | 2.03 |
|  |  | (n=6) | (n=7) | (n=7) | (n=4) |  |  |
| Expression of each gene regarding to X-inactivation pattern and intelligence quotient, showing that individuals 47,XXY with SXI present gene expression level similar to those 46,XY controls. Data are expressed in relation to male controls and represent mean ± SEM of experiments performed with the indicated number of individuals. Statistical analysis are not shown. RXI, random X-inactivation; SXI, skewed X-inactivation. | | | | | | | |
